# Association between obstructive sleep apnea symptoms and gout in US population, a cross-sectional study

**DOI:** 10.1038/s41598-023-36755-4

**Published:** 2023-06-23

**Authors:** Xi Gu, Dou Tang, Yan Xuan, Ying Shen, Lei Qun Lu

**Affiliations:** Department of Endocrinology, RuiJin Hospital Lu Wan Branch, Shanghai Jiaotong University School of Medicine, No.149 Chongqing South Road, Shanghai, China

**Keywords:** Respiratory signs and symptoms, Crystal deposition arthropathies

## Abstract

The results of association between Obstructive Sleep Apnea (OSA) and gout are not consistent. Participants aged 20 years or older in the National Health and Nutrition Examination Survey (NHANES) 2007–2008 and 2015–2018 were included. Weighted univariable and multivariable logistic regressions were used to evaluate the association between OSA symptoms and gout. The subgroup and sensitivity analyses were also performed. Among the 15,947 participants in this study, the mean age was 47.8 years old, 48.87% of whom were male, 4891 had OSA symptoms, and 842 had gout. In multivariable logistic regression analyses, OSA symptoms were positively associated with gout in all models. The odds ratio (OR) was 1.315 and 95% confidence interval (CI) was 1.070–1.616 in fully adjusted model 4. In the subgroup analyses, we found a considerable interaction between OSA symptoms and gender with gout (*P* for interaction = 0.003). In the sensitivity analyses, the association between OSA symptoms and gout remained stable after adjustment for congestive heart failure and diuretics using. OSA symptoms were associated with an increased likelihood of gout. This association could especially be found in female participants.

## Introduction

Obstructive Sleep Apnea (OSA) is a common problem that disrupts breathing during sleep. Symptoms of OSA include loud, frequent, bothersome snoring; witnessed episodes of apnea; choking/gasping during sleep; excessive daytime sleepiness etc.^1^ It increases risk for motor vehicle and occupational accidents^2^. Approximately 25% of adults in the US have been found to have OSA^3^. Between 1990 and 2010, the prevalence of OSA saw an increase of about 30%. Men experienced an absolute increase of 7.5%, while women saw an increase of 4.2%^4^. Untreated OSA is associated with a series of adverse consequences, like metabolic disorders, cardiovascular and cerebrovascular diseases^5–7^, which seriously threaten public health.

Gout is a form of arthritis caused by the buildup of uric acid crystals in the body. It affects about 3.9% adult population in the US^8^. Obesity, hypertension, and chronic kidney disease are the most common comorbidities associated with gout in the US^9^. Moreover, a published study reported a higher prevalence of gout in males, older adults, and non-Hispanic whites^10^. Commonly, a gout flare occurs over several hours and causes severe pain, redness and swelling in single or multiple joints. If gout were not treated properly, it would cause permanent damage to the joints and form tophi. A study found that gout flares were associated with a transient increase in cardiovascular events following the flare^11^.

Although some studies^12,13^ have investigated the relationship between OSA and gout, the relationship has not been assessed completely in recent years in the US. Neither the home sleep apnea testing nor the in-laboratory polysomnography is largely used, the numbers of OSA patients are likely to be underestimated^14^. Furthermore, the effect of OSA on gout may be overlooked.

Therefore, we conducted a cross-sectional study, using the National Health and Nutrition Examination Survey (NHANES) database, to help us determine the association between OSA symptoms and gout. We hypothesized that the symptoms of OSA may be associated with the occurrence of gout even after adjustment for confounding factors.

## Methods

### Data sources

NHANES is a cross-sectional, nationally representative survey of the non-institutionalized civilian population of the United States. It is conducted annually by the centers for disease control and prevention’s national center for health statistics (CDC/NCHS).

During the in-person interviews, participants reported demographic and in-depth health information to the NCHS-trained professionals in their homes. After a home interview, participants were invited to attend examination sessions in the mobile examination center (MEC). Physical and laboratory examinations, including blood and urine collection, were conducted at MEC. The details of the programs, collection procedures and data files are publicly available at http://www.cdc.gov/nchs/nhanes.html.

All procedures in each NHANES were approved by the national center for health statistics ethics review board, and written informed consent was obtained from participants at the time of enrollment. This study followed the strengthening the reporting of observational studies in epidemiology (STROBE) reporting guideline for cross-sectional studies.

### Study design and population

**Figure 1:**
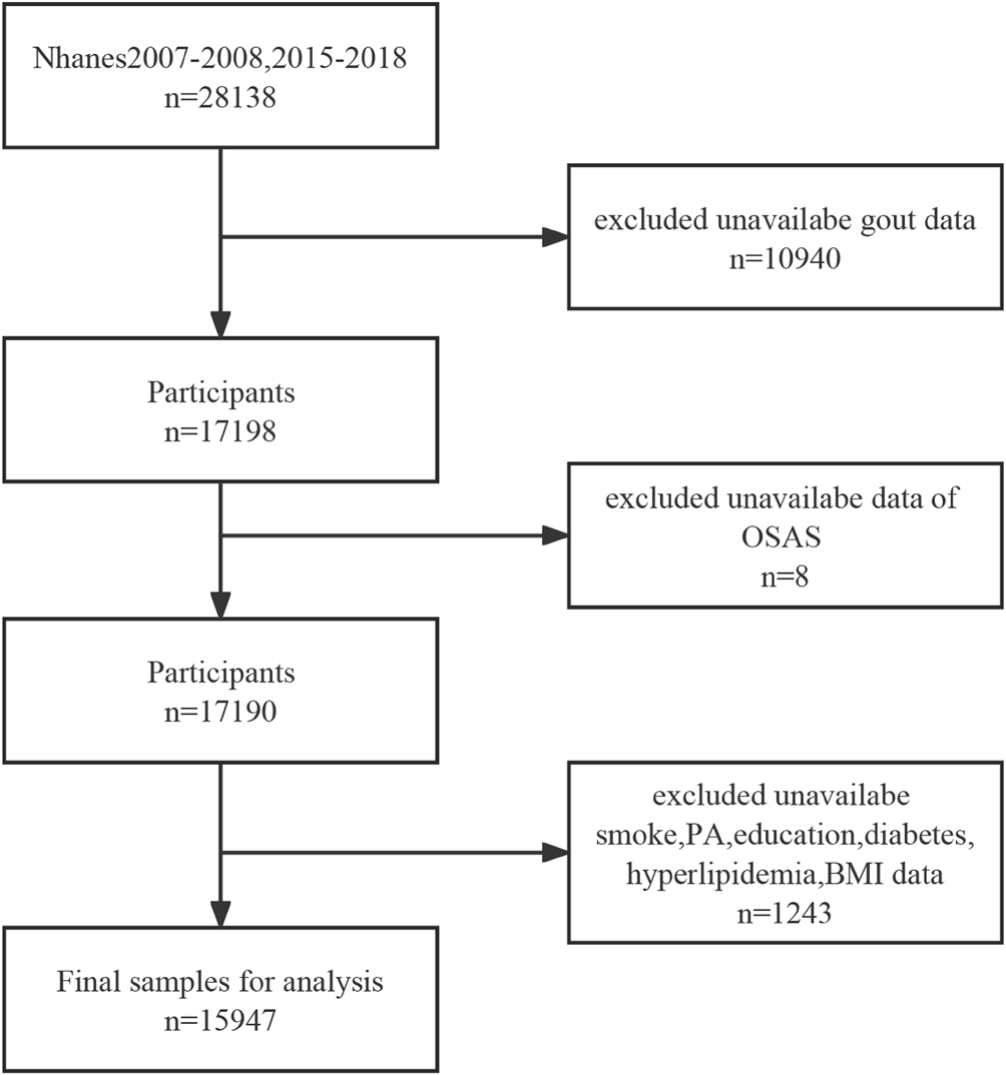
Flowchart of the study design. *NHANES* National Health and Nutrition Examination survey, *OSAS* obstructive sleep apnea symptoms, *BMI* Body Mass Index, *PA* physical activity.

For our study, we used data from the NHANES 2007–2008, 2015–2018-year circles. A total of 28,138 participants aged 20 years or older constituted the initial sample. After excluding individuals with missing data for OSA symptoms, gout, smoke, physical activity (PA), education, diabetes, hyperlipidemia, and body mass index (BMI), 15,947 participants (7793 men and 8154 women) were included in our final analyses (see Fig. 1).

### Definition of OSA symptoms

OSA symptoms were defined based on answers to three dichotomous questions^15^. They include (1) how often you snore; (2) how often you snort/stop breathing; or (3) how often you feel overly sleepy during day. Individuals who answered that they snore 3 or more per week; snort/stop breathing 3 or more per week and feel overly sleepy during day 16–30 times per month were classified as having OSA symptoms.

### Definition of gout

The presence of gout was obtained from medical condition questionnaire, based on the self-reported answer to: “Has a doctor or other health professional ever told you that you have gout?”. If the answer was yes, the participant was defined as having gout.

### Covariates

The covariates, which were selected on the basis of previous research^12,13,16,17^ and clinical judgment included age, gender, ethnicity, BMI, poverty–income ratio (PIR), total meat consumption, alcohol consumption, estimated glomerular filtration rate (eGFR), educational level, diabetes, hypertension, hyperlipidemia, PA and smoking status. BMI is calculated as weight in kilograms divided by height in meters squared. PIR is calculated by the family size-specific threshold. Total meat consumption and alcohol consumption was extracted from Food Patterns Equivalents Database (FPED) diet data, the unit is oz/d and drink/d respectively. eGFR was estimated by using the 2009 CKD-EPI formula^18^. Ethnicity was categorized as non-Hispanic white, non-Hispanic black, Mexican American, or other races. Education status was classified as less than high school, high school, or college. The definition of diabetes was based on the American Diabetes Association criteria and a self-report questionnaire. Participants who fulfilled the following criteria were identified as diabetes cases: (1) Fasting blood sugar (FBS) ≥ 7 mmol/L, (2) HbA1c ≥ 6.5%, (3) 2-h plasma glucose ≥ 11.1 mmol/L during an oral glucose tolerance test, (4) self-report questionnaire data indicating physician diagnosis of diabetes, and current use of insulin or diabetes pill to lower blood glucose. The presence of hypertension was defined by using blood pressure measurements during physical examination (systolic blood pressure ≥ 140 mm Hg or diastolic blood pressure ≥ 90 mm Hg) or the responses to interview questions about being told of having high blood pressure or taking blood pressure medications. Hyperlipidemia was diagnosed based on the participant's total cholesterol ≥ 200 mg/dl, LDL ≥ 130 mg/dl, HDL < 40 mg/dl(male)/50 mg/dl(female) or triglycerides ≥ 150 mg/dl, or the lipid-lowering medication status. Smoking status was categorized as never smokers (smoked less than 100 cigarettes), former smokers (quit smoking after smoking more than 100 cigarettes) and current smokers. PA was determined by whether the individuals participated in the walking or bicycling. Age, gender, ethnicity, PIR, educational level, PA, and smoking status were collected from the questionnaire. Total meat and alcohol consumption were collected from the dietary data. eGFR was obtained from the laboratory data.

### Statistical analysis

Data analysis was performed from August to November 2022. Both descriptive and regression analyses were performed by using weighted samples. Continuous variables were expressed as mean and standard error (SE) and categorical variables were expressed as weighted percentages (%) in descriptive analysis. Student’s t-test was used to compare the mean levels between the with-gout group and the without-gout group if the variable was normally distributed. Chi-square tests were chosen to compare the percentages of categorical variables between the different groups. Univariate and multivariate binary logistic regression model were used to test the link between OSA symptoms and gout. The association between OSA symptoms and gout was measured using odds ratio (OR). All confidence intervals (CI) were 95%. In model 1, we did not adjust for any confounders. In model 2, we adjusted for age, gender, and ethnicity. In model 3, we additionally adjusted for PIR, BMI, total meat consumption, alcohol consumption, smoking status, PA. In model 4, we additionally adjusted for diabetes, hypertension, hyperlipidemia and eGFR.

The aim of the subgroup analyses was to assess whether there were potential effect modifications on the relationship between OSA symptoms and gout. We did the analyses by the following variables: age (< 65and ≥ 65), gender, BMI (< 30and ≥ 30), eGFR (< 60and ≥ 60), diabetes, hypertension, hyperlipidemia. Subgroup analyses were performed using stratified logistic regression models. The modifications and interactions of subgroups were inspected by likelihood ratio tests. Each stratification adjusted for the factors (age, ethnicity, PIR, BMI, meat consumption, alcohol consumption, smoking status, PA, eGFR).

To assess the robustness of the findings, two sensitivity analyses were conducted. First, congestive heart failure (HF) as a confounder was additionally adjusted. HF was determined by the question “has a doctor or other health professional ever told you that you had congestive heart failure?”. Second, diuretics using as a confounder was adjusted. Diuretics included furosemide or hydrochlorothiazide.

The missing values of covariate (PIR, total meat consumption, and alcohol consumption) were replaced by the mean values.

All the analyses were performed with the statistical software packages R 4.2.1 (http://www.R.project.org, The R Foundation) and Free Statistics software versions 1.7.1. *P*-value < 0.05 (two-sided) was considered statistically significant.

### Ethics approval and consent to participate

All NHANES protocols were approved by the ethics review board of the National Center for Health Statistics. All participants have written informed consents. All methods were carried out in accordance with relevant guidelines and regulations.

## Results

### Baseline characteristics of the study population

Based on the weighted analyses, baseline characteristics of the15947 enrolled participants stratified by gout status are shown in Table 1. There were 842 (5.28%) individuals with gout and 4891 (30.67%) with OSA symptoms. The prevalence of gout was higher among participants with OSA symptoms (331 [6.76%]) than those without OSA symptoms (511 [4.62%]). The average age was 47.8 (0.31) years and 7793 (48.87%) were male. BMI and eGFR of the study participants were 29.27 (0.14) kg/m^2^ and 94.61 (0.46) ml/min. Individuals in the gout group were predominated by male participants, former smoker, and non-Hispanic White. In addition, participants with gout were more likely to be older, obese, non-diabetes individuals and to have hypertension, hyperlipidemia, less PA frequency and lower eGFR.

**Table 1:** Baseline characteristics of participants in the NHANES 2007–2008 and 2015–2018 cycles.

Variable	Total	Without gout (n = 15,105)	Gout (n = 842)	*P* value
Age (years)	47.80 (0.31)	47.19 (0.32)	61.51 (0.66)	< 0.0001
BMI (kg/m^2^)	29.27 (0.14)	29.15 (0.13)	31.92 (0.38)	< 0.0001
PIR	2.99 (0.04)	2.99 (0.05)	2.90 (0.09)	0.25
Meat (oz/day)	4.89 (0.05)	4.88 (0.05)	5.09 (0.25)	0.39
Alcohol (drink/day)	0.79 (0.03)	0.79 (0.03)	0.85 (0.13)	0.65
eGFR (ml/min)	94.61 (0.46)	95.41 (0.48)	76.63 (0.88)	< 0.0001
Gender				< 0.0001
Female	8154 (51.13)	7903 (52.29)	251 (31.81)	
Male	7793 (48.87)	7202 (47.71)	591 (68.19)	
Ethnicity				0.004
Non-Hispanic White	6118 (38.36)	5748 (65.19)	370 (69.13)	
Other	3843 (24.1)	3670 (14.88)	173 (13.58)	
Mexican	2531 (15.87)	2456 (8.75)	75 (4.44)	
Non-Hispanic Black	3455 (21.67)	3231 (11.18)	224 (12.86)	
Education				0.46
Less than high school	3968 (24.88)	3747 (15.06)	221 (16.62)	
High school	3744 (23.48)	3544 (24.39)	200 (25.66)	
College	8235 (51.64)	7814 (60.55)	421 (57.71)	
Diabetes				< 0.0001
No	12,698 (79.63)	12,220 (85.82)	478 (64.00)	
Yes	3249 (20.37)	2885 (14.18)	364 (36.00)	
Hypertension				< 0.0001
No	8875 (55.65)	8705 (63.23)	170 (22.21)	
Yes	7072 (44.35)	6400 (36.77)	672 (77.79)	
Hyperlipidemia				< 0.0001
No	5030 (31.54)	4870 (33.32)	160 (20.16)	
Yes	10,917 (68.46)	10,235 (66.68)	682 (79.84)	
PA				0.003
No	12,217 (76.61)	11,525 (78.48)	692 (84.65)	
Yes	3730 (23.39)	3580 (21.52)	150 (15.35)	
Smoking status				< 0.0001
Never	8925 (55.97)	8565 (56.16)	360 (43.02)	
Former	3826 (23.99)	3473 (24.01)	353 (43.20)	
Current	3196 (20.04)	3067 (19.83)	129 (13.78)	
OSA symptoms				< 0.0001
No	11,056 (69.33)	10,545 (69.52)	511 (57.59)	
Yes	4891 (30.67)	4560 (30.48)	331 (42.41)	

*NHANES* National Health and Nutrition Examination Survey, *BMI* body mass index, *PIR* poverty income ratio, *PA* physical activity, *OSA* obstructive sleep apnea, *eGFR* estimated glomerular filtration rate.

### Relationship between OSA symptoms and gout

Based on the sample-weighted analyses, we performed the univariate logistics regression (Table S1). The results showed that having OSA symptoms, age, male, Mexican American (compared with non-Hispanic White individuals), BMI, former smoker (compared with never smoking individuals), taking PA, eGFR, diagnosed diabetes, hypertension and hyperlipidemia were associated with gout (*P*-value < 0.05).

The results of sample-weighted multivariable logistics regression analyses were presented in Table 2. The positive association between OSA symptoms and gout was revealed in all models. The OR and 95% CI were 1.68 (1.393 ~ 2.025), 1.56 (1.286 ~ 1.897), 1.35 (1.102 ~ 1.654) and 1.315 (1.07 ~ 1.616), respectively from model 1 to model 4.

**Table 2:** Association between OSA symptoms and gout in weighted multivariable logistic regression.

Variable	Model 1		Model 2		Model 3		Model 4	
	OR (95% CI)	*P* value	OR (95% CI)	*P* value	OR (95% CI)	*P* value	OR (95% CI)	*P* value
OSAS no	1 (ref)		1 (ref)		1 (ref)		1 (ref)	
OSAS yes	1.68 (1.393–2.025)	< 0.0001	1.56 (1.286–1.897)	< 0.0001	1.35 (1.102–1.654)	0.005	1.315 (1.07–1.616)	0.011

*OSAS* obstructive sleep apnea symptoms, *BMI* body mass index, *PIR* poverty income ratio, *eGFR* estimated glomerular filtration rate, *PA* physical activity, *OR* odds ratio, *CI* confidence interval, *Ref* reference.

Model 1: crude model.

Model 2: adjusted for age, gender, ethnicity.

Model 3: adjusted for age, gender, ethnicity, PIR, BMI, meat consumption, alcohol consumption, smoking status, PA.

Model 4: adjusted for age, gender, ethnicity, PIR, BMI, meat consumption, alcohol consumption, smoking status, PA, diabetes, hypertension, hyperlipidemia, eGFR.

### Subgroup analyses

**Figure 2:**
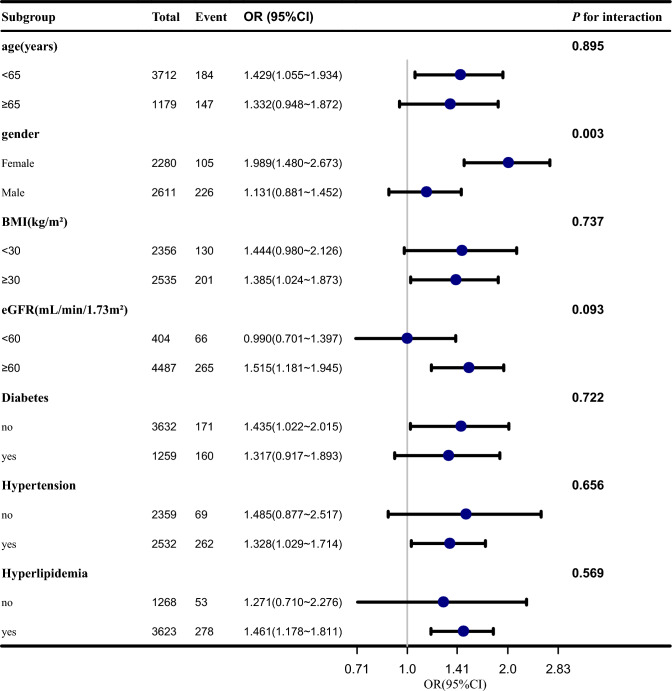
Multivariable odds ratio (OR) for gout status based on OSA symptoms stratified by age, gender, BMI, Diabetes, Hypertension, Hyperlipidemia and eGFR. Each stratification adjusted for the factors (age, ethnicity, PIR, BMI, meat consumption, alcohol consumption, smoking status, PA, eGFR). *OSA* obstructive sleep apnea, *BMI* Body Mass Index, *eGFR* estimated glomerular filtration rate, *PIR* poverty income ratio, *PA* physical activity, *OR* odds ratio, *CI* Confidence interval.

The results of subgroup analyses were presented in Fig. 2. We found a considerable interaction between OSA symptoms and gender with the risk of gout (*P* for interaction = 0.003). In the female subgroup, OSA symptoms was associated with an increased risk of gout (OR 1.989;95% CI 1.480–2.673), while in the male subgroup compared with the reference group (without OSA symptoms), the OR was 1.131 (95% CI 0.881–1.452).No other significant interactions were found in the subgroups stratifying by age, BMI, eGFR, diagnosed diabetes, hypertension and hyperlipidemia.

### Sensitivity analysis

In the sensitivity analysis, HF was additionally adjusted, OSA symptoms was associated with gout (OR 1.32, 95% CI 1.07–1.62). After additionally adjusted for diuretics using, the association was remained (OR 1.31, 95% CI 1.07–1.62). The results were presented in Table 3.

**Table 3:** Sensitivity analysis of association between OSA symptoms and gout.

Variable	Model 1		Model 2		Model 3	
	OR (95%CI)	*P* value	OR (95%CI)	*P* value	OR (95%CI)	*P* value
OSAS no	1 (ref)		1 (ref)		1 (ref)	
OSAS yes	1.68 (1.393–2.025)	< 0.0001	1.32 (1.07–1.62)	0.01	1.31 (1.07–1.62)	0.01

*OSAS* obstructive sleep apnea symptoms, *BMI* body mass index, *PIR* poverty income ratio, *eGFR* estimated glomerular filtration rate, *PA* physical activity, *HF* heart failure, *OR* odds ratio, *CI* confidence interval, *Ref* reference.

Model 1: crude model.

Model 2: adjusted for age, gender, ethnicity, PIR, BMI, meat consumption, alcohol consumption, smoking status, PA, Diabetes, Hypertension, Hyperlipidemia, eGFR, HF.

Model 3: adjusted for age, gender, ethnicity, PIR, BMI, meat consumption, alcohol consumption, smoking status, PA, Diabetes, Hypertension, Hyperlipidemia, eGFR. diuretics using.

## Discussion

We conducted a cross-sectional study in the US population with data from the NHANES database, and the results showed that there was a positive association between OSA symptoms and gout. This positive correlation persisted after adjustment for covariates, such as age, gender, ethnicity, PIR, BMI, meat consumption, alcohol consumption, smoke, diabetes, hypertension, hyperlipidemia, PA and eGFR. We performed both subgroup and sensitivity analyses, and the results showed that the association between OSA symptoms and gout remained stable.

A previous meta-analysis^19^, comprising three UK-based studies and one from Taiwan, China, found a positive association between OSA and gout that did not reach statistical significance. The analysis revealed a 25% increase in the likelihood of gout with OSA (HR 1.25; 95% CI 0.91–1.70), indicating the need for further investigations to establish whether OSA is indeed a risk factor for gout. As such, considering the incomplete survey of the association and insufficient evidence from the US, our cross-sectional study on a general US population serves as an important addition to the existing knowledge base, distinct from the aforementioned meta-analysis. There are some published studies, their conclusions were not consistent with ours completely. A case–control study was conducted in 2020^13^, its data were obtained from the Clinical Practice Research Datalink (CPRD) in the UK, and all the participants were older than 40 years. This study included HF as a significant covariate, because previous studies have suggested that HF is associated with both gout^20^ and with sleep disorders^21^, especially OSA. This study showed that the observed association between OSA and gout disappeared after adjusting for BMI, HF, diuretics, and renal function particularly. The diagnosis of HF in the study is determined through the diagnostic codes, which might lead to some under-diagnosis^13^. In our study, HF was determined by questionnaire(has a doctor or other health professional ever told you that you had congestive heart failure?). In sensitivity analysis, we added HF as a covariate for adjusted, and the association between OSA symptoms and gout did not disappear (Table 3). Another cohort study in 2019^16^, using the same UK population (CPRD), found that people with OSA continued to be at higher risk of developing gout beyond the first year after OSA diagnosis. The median follow-up was 5.8 years. Interestingly, the risk is not same in different BMI groups. Those who had normal BMI was at greater risk of gout than those who were overweight or obese. This suggests that clinical physicians should consider the possibility of gout in patients with sleep apnea regardless of obesity. Similarly, in our study, a stratified analysis showed that OSA symptoms seemed more strongly associated with gout in the people who were not obese than those who were obese (OR 1.750 and 1.313), but the result did not reach statistical difference. In 2018, an observational study^17^ investigated gout and the risk of incident obstructive sleep apnea in adults 65 years or older during the period 2006–2012.Their study also supported the positive association between OSA and gout. After adjusted for demographics, age, sex, race, Charlson-Romano comorbidity score, hypertension, hyperlipidemia and coronary artery disease, there was an independent association of gout with a twofold higher risk of OSA in older adults. This study included a large population but lacked diet-related data and was unable to control for obesity due to the lack of BMI data. So, the conclusion may have a limitation, because we know high purine diets are associated with hyperuricemia as well as gout^22^. Our study incorporated dietary data on meat, seafood, etc., and we studied a wider age range of the population in the US. In 2015, this positive correlation was also found in a British study^12^. They identified 270 incident cases of gout over 1 year of follow-up, so they had fewer variables included in the stratified analysis. Our study had more gout events, and there were more variables in the stratified analysis, such as diabetes, hypertension, hyperlipidemia, etc.

In some studies, the potential mechanisms about the relationship between OSA and gout were discussed. First, hypoxemia induced by OSA is the reason that can cause a rise in adenosine triphosphate (ATP) degradation, which further increases purine concentrations and their end product uric acid^23^. Second, OSA can cause hypercapnia and acidosis, which could increase the likelihood of monosodium urate precipitation^24^. Third, the excretion of lactate produced during hypoxic episodes of OSA may lead to increased renal reabsorption of uric acid, and further lead to gout flare^25^.

Uric acid is produced from the breakdown of purines, which are found in many foods^22^ and are generated during the metabolism of nucleic acids^26^. High levels of uric acid in the blood, known as hyperuricemia, increase the likelihood of monosodium urate (MSU) crystal deposition in joints, tendons, and other tissues^27^. The MSU crystals activate the innate immune system, resulting in the release of proinflammatory cytokines and chemokines, ultimately leading to the inflammatory response associated with gout attacks^28^. Recent studies have elucidated the mechanisms through which MSU crystals cause inflammation, including the activation of the NLRP3 inflammasome^29^ and subsequent release of IL-1β, a potent proinflammatory cytokine^30^. The MSU crystals also stimulate neutrophils to produce reactive oxygen species (ROS) and release extracellular DNA, further contributing to the inflammatory response^31^.

Our study has some strengths. To our knowledge, few similar studies have been conducted in the US population. Because the weighted samples analyses were used to explore the association, the results could be generalizable to the population of US adults. In addition, compared with other studies, we adjusted for dietary variables more adequately in our multivariate regression analysis. At last, we performed a sensitivity analysis, and the results suggested that the association remained stable.

There were several limitations in our study. We could only find some of the most typical OSA symptoms through the sleep questionnaire, such as snoring, stop breathing, and daytime sleepy, while other symptoms such as morning headache, and driving accidents could not be obtained from the NHANES database. As the same reason, the respondents did not undergo laboratory sleep testing, or home sleep apnea testing^1^, so maybe some of the individuals with OSA were not diagnosed. But there have been some studies about OSA symptoms using NHANES database^15,32^. The diagnosis of gout was made by a questionnaire (whether you have ever been told by a doctor or health professional that you have gout), so perhaps some respondents with a history of joint pain did not go to the hospital and therefore do not know whether they had a gout flare. However, such misclassification is likely to be nondifferential^12^. At last, we conducted a cross-sectional survey and were unable to draw causal conclusions.

## Conclusions

This cross-sectional study suggested a positive association between OSA symptoms and the incidence of gout. More research is needed to investigate whether treatments targeting OSA, such as continuous positive airway pressure (CPAP), can reduce the incidence of gout.

## Supplementary Information

Supplementary Table S1.

## Data Availability

All the datasets collected and analyzed during this study are available on the NHANES website (http://www.cdc.gov/nchs/nhanes.htm).

## Abbreviations

OSA: Obstructive sleep apnea
BMI: Body mass index
PIR: Poverty income ratio
PA: Physical activity
eGFR: Estimated glomerular filtration rate
NHANES: National health and nutrition examination survey
CDC: Centers for disease control
NCHS: National center for health statistics
MEC: Mobile examination center
FPED: Food patterns equivalents database
HF: Heart failure
CPAP: Continuous positive airway pressure
OR: Odds ratio
CI: Confidence interval
Ref: Reference
