# Supplementary material for: Association between obstructive sleep apnea symptoms and gout in US population, a cross-sectional study
Source: Sci Rep. 2023 Jun 23;13:10192. doi: 10.1038/s41598-023-36755-4 (PMC10290056; doi:10.1038/s41598-023-36755-4)
Supplement: Supplementary file 1 — Supplementary Table S1. [file 41598_2023_36755_MOESM1_ESM.docx]

**Table S1** Association between univariable and gout in logistic regression analysis

| Variable | OR (95%CI) | *P* value |
| --- | --- | --- |
| OSA symptoms no | Ref |  |
| yes | 1.68 (1.39~2.03) | <0.0001 |
| Age(years) | 1.05 (1.05~1.06) | <0.0001 |
| Gender Female | Ref |  |
| Male | 2.35 (1.88~2.93) | <0.0001 |
| Ethnicity Non-Hispanic White | Ref |  |
| Mexican | 0.48 (0.35~0.66) | <0.0001 |
| Other | 0.86 (0.63~1.18) | 0.35 |
| Non-Hispanic Black | 1.08 (0.86~1.37) | 0.48 |
| Education Less than High school | Ref |  |
| High school | 0.95 (0.72~1.27) | 0.74 |
| College | 0.86 (0.67~1.11) | 0.24 |
| BMI (kg/m2) | 1.05(1.04~1.06) | <0.0001 |
| PIR | 0.96 (0.91~1.03) | 0.25 |
| Smoke never | Ref |  |
| former | 2.35 (1.85~2.99) | <0.0001 |
| current | 0.91 (0.62~1.33) | 0.61 |
| Alcohol(drink/d) | 1.02 (0.95~1.08) | 0.62 |
| Meat(oz/d) | 1.01 (0.98~1.04) | 0.37 |
| PA no | Ref |  |
| yes | 0.66 (0.51~0.86) | 0.003 |
| eGFR (ml/min) | 0.96 (0.96~0.97) | <0.0001 |
| Diabetes no | Ref |  |
| yes | 3.4 (2.75~4.22) | <0.0001 |
| Hypertension no | Ref |  |
| yes | 6.02(4.73~7.66) | <0.0001 |
| Hyperlipidemia no | Ref |  |
| yes | 1.98 (1.53~2.56) | <0.0001 |

OSA: obstructive sleep apnea; BMI: body mass index; PIR: poverty income ratio.

eGFR: estimated glomerular ﬁltration rate; PA: physical activity.

OR: odds ratio; CI: confidence interval；Ref: reference.
